# Blood flow-bearing physical forces, endothelial glycocalyx, and liver enzyme mobilization: A hypothesis

**DOI:** 10.1085/jgp.202313462

**Published:** 2024-01-17

**Authors:** Lorena Carmina Hernández-Espinosa, Rolando Hernández-Muñoz

**Affiliations:** 1 Department of Cell Biology and Development, Institute of Cellular Physiology, Universidad Nacional Autónoma de México (UNAM), Mexico City, Mexico

## Abstract

Numerous elements involved in shear stress-induced signaling have been identified, recognizing their functions as mechanotransducing ion channels situated at cellular membranes. This form of mechanical signaling relies on transmembrane proteins and cytoplasmic proteins that restructure the cytoskeleton, contributing to mechanotransduction cascades. Notably, blood flow generates mechanical forces that significantly impact the structure and remodeling of blood vessels. The primary regulation of blood vessel responses occurs through hemodynamic forces acting on the endothelium. These mechanical events intricately govern endothelial biophysical, biochemical, and genetic responses. Endothelial cells, positioned on the intimal surface of blood vessels, have the capability to express components of the glycocalyx. This endothelial structure emerges as a pivotal factor in mechanotransduction and the regulation of vascular tone. The endothelial glycocalyx assumes diverse roles in both health and disease. Our findings propose a connection between the release of specific enzymes from the rat liver and variations in the hepatic blood flow/mass ratio. Importantly, this phenomenon is not correlated with liver necrosis. Consequently, this review serves as an exploration of the potential involvement of membrane proteins in a hypothetical mechanotransducing phenomenon capable of controlling the release of liver enzymes.

## Teaser

The mechanisms governing the release of hepatic enzymes are largely unknown. It is hypothesized that hemodynamic changes, exerting mechanotransducing actions on the liver endothelium, play a crucial role in stimulating the release of enzymes from the intact liver.

## Introduction

Mechanotransduction is a process that initiates biochemical signaling in response to mechanical events, enabling cells to interact with and adapt to their physical environment. Mechanosensitive factors play a crucial role in this process. Plasma membrane stretch-activated ion channels, such as those discussed by Wang et al. (2009), induce alterations in the cytoskeleton. Additionally, nuclear lamins, acting as connectors between the nucleus and the cytoskeleton, undergo changes under mechanical stimuli (Zwerger et al., 2013).

**Figure 1.**
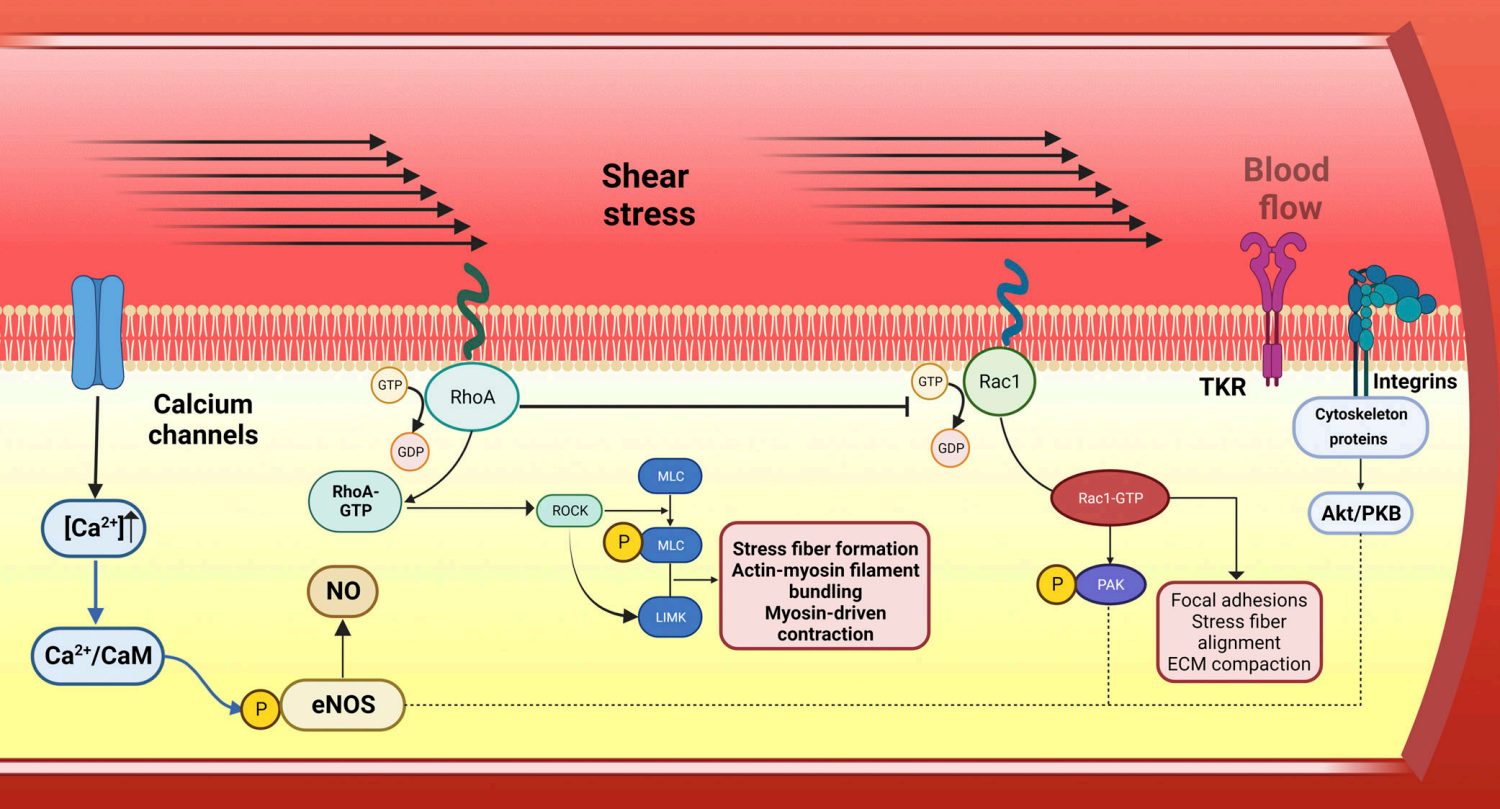
**Mechanotransduction of the vascular response.** Blood flow through arteries and shear stress initiate the activation of mechanosensors, such as the activation of RhoA protein and further activation of Rac1, which controls cytoskeletal reorganization by regulating actin metabolism. RhoA and Rac1 constitute a subgroup of the Ras superfamily of GTP hydrolases. ROCK, an effector molecule of RhoA, increases the phosphorylation of MLC of myosin II and LIMK among other pathways. Rac1-GTP activates PAK, resulting in PAK1 autophosphorylation. Other endothelial mechanosensors like TKR and integrins can connect the extracellular matrix with the actin cytoskeleton, activating the Akt/PKB signaling pathway. The activation of ion channels leads to short-term regulation of eNOS by an increase in intracellular calcium concentration and/or by activation of protein kinases. Abbreviations: CaM, calmodulin; eNOS, endothelial nitric oxide synthase; PAK, p21-regulated kinase; ROCK, Rho-kinase; MLC, myosin light chain; LIM-kinase; TKR, tyrosine kinase receptor; and PKB, protein kinase B.

Cell signaling induced by mechanical stimuli involves various mechanisms. Stretch-gated ion channels, as highlighted by Sukharev and Sachs (2012), can be activated, and other proteins undergo conformational changes, leading to bond formation, alterations in phosphorylation states, or changes in protein stability (Iskratsch et al., 2014). For instance, talin, a focal adhesion protein, unfolds under stress, revealing vinculin-binding sites (Del Río et al., 2009). Cell junction proteins, like those described by Borghi et al. (2012), transmit stresses between adherent cells. At the molecular level, external perturbations like shear, compressive, or tensile stress modulate contractility by regulating RhoA activity and its kinase (Watanabe et al., 1999; Lessey et al., 2012; Fig. 1). Integrins contribute to RhoA activation by recruiting RhoGEFs GEF-H1 and LARG to focal adhesions (Guilluy et al., 2011; Fig. 1).

Mechanical forces significantly impact the regulation of cellular functions (Mammoto et al., 2009; Weber et al., 2012). The cell’s mechanical environment comprises both endogenous forces, generated by the cell’s tension on the extracellular matrix, and exogenous forces, such as shear stress and hydrostatic pressure from external sources like blood flow. Coordinating these events involves the activation of multiple signaling cascades (Matthews et al., 2006; Collins et al., 2012). Moreover, PECAM-1 initiates various intracellular signaling pathways in response to hemodynamic forces, and integrin heterodimers bind to specific extracellular matrix proteins within the endothelium (Tzima et al., 2005; Orr et al., 2006).

The endothelium plays a crucial role in maintaining the delicate balance between coagulation and fibrinolysis, among other hematological functions. The primary physiological event facilitating endothelial cell regulation of blood flow is the tangential shear stress generated by the blood flow. Endothelial cells are connected toward the vessel lumen for this purpose, and shear stress is influenced by factors like flow rate, blood viscosity, and specific tube geometry (Humphrey et al., 2009; Hoefer et al., 2013).

In addition to intravascular flow, interstitial (transvascular) flow potentially contributes to angiogenesis (Kutys and Chen, 2016). The endothelial glycocalyx plays a crucial role in regulating vascular permeability, transmitting shear stress, and modulating vascular homeostasis, including nitric oxide (NO) production and vasodilation (Cao et al., 2019).

Mechanical forces, particularly cyclic stretch, play significant roles in regulating vascular functions across various organs, including the maintenance of pulmonary circulation homeostasis (Birukov, 2009; Ando and Yamamoto, 2011). The liver sinusoidal endothelium is structurally and functionally unique in multiple ways (Birukova et al., 2005; Glass and Natoli, 2016). The multifaceted roles of the endothelial glycocalyx in both health and disease lead to the hypothesis that the liver endothelial glycocalyx can regulate the release of specific enzymes from the rat liver, depending on changes in the hepatic blood flow/mass ratio, without being associated with liver necrosis.

## Hemodynamic forces

Hemodynamic forces acting on the vessel wall are ubiquitous stimuli for vascular cells, and the mechanical signals emitted by the microenvironment play crucial regulatory roles in controlling cell behavior. This occurs under a diverse spectrum of hemodynamic stresses that exhibit significant variations in physical properties (Dewey, 1984; Kim et al., 1989). Hemodynamic forces, particularly fluid shear stress, profoundly impact the turnover of endothelial cells and the regulation of various molecular factors. These forces influence the expression and release of growth factors, cytokines, adhesion molecules, vasodilators, vasoconstrictors, and reactive oxygen species (ROS), thereby influencing the immune system’s interaction with endothelial cells (Cahill and Redmond, 2016). In addition, the presence of low shear stress plays a significant role in facilitating interactions between erythrocytes and endothelial von Willebrand factor (VWF), as well as between erythrocyte–VWF and fibrin. These interactions directly contribute to the development of venous thrombosis (Smeets et al., 2017).

The flow of blood in the arterial circulation is pulsatile, exhibiting its effects on the physical features of blood vessels through perpendicular and parallel vectors (Dewey, 1984; Nerem, 1992). Although the fibrillar protein framework of the extracellular matrix is subject to stretching, shear stress has a predominant impact on endothelial cells. The malleability of cells in response to flow forces results in an aligned shear stress (Yoshida et al., 1990). This capability allows endothelial cells to actively modify their structure and mechanical properties, leading to the generation of intracellular stress. For instance, cytoskeletal reorganization can occur as a response to flow.

## Shear stress and mechanical stretch

Physiologically, the straight sections of blood vessels are subject to laminar flux (shear stress), characterized by unidirectional orientation flow. It plays various roles, including anti-inflammation, anticoagulation, antioxidant, and antiapoptotic functions, crucial for maintaining endothelial cells in a quiescent phenotype. The orientation of endothelial cells and the direction of blood flow have been documented since early observations (Flaherty et al., 1972; Davies et al., 1976; Okano and Yoshida, 1993). The cytoskeleton indeed determines cell shape and orientation as it undergoes rearrangements due to the flow.

Furthermore, mechanical stretch acts on vascular development, smooth muscle differentiation, aneurysm formation, and the progression of hypertension (Milewicz et al., 2017). Specifically, physiological mechanical stretch is essential for maintaining vascular homeostasis by regulating cellular structure, promoting vascular angiogenesis, and modulating vessel tone (Ando and Yamamoto, 2011).

## Endothelial glycocalyx: Structure and functions

**Figure 2.**
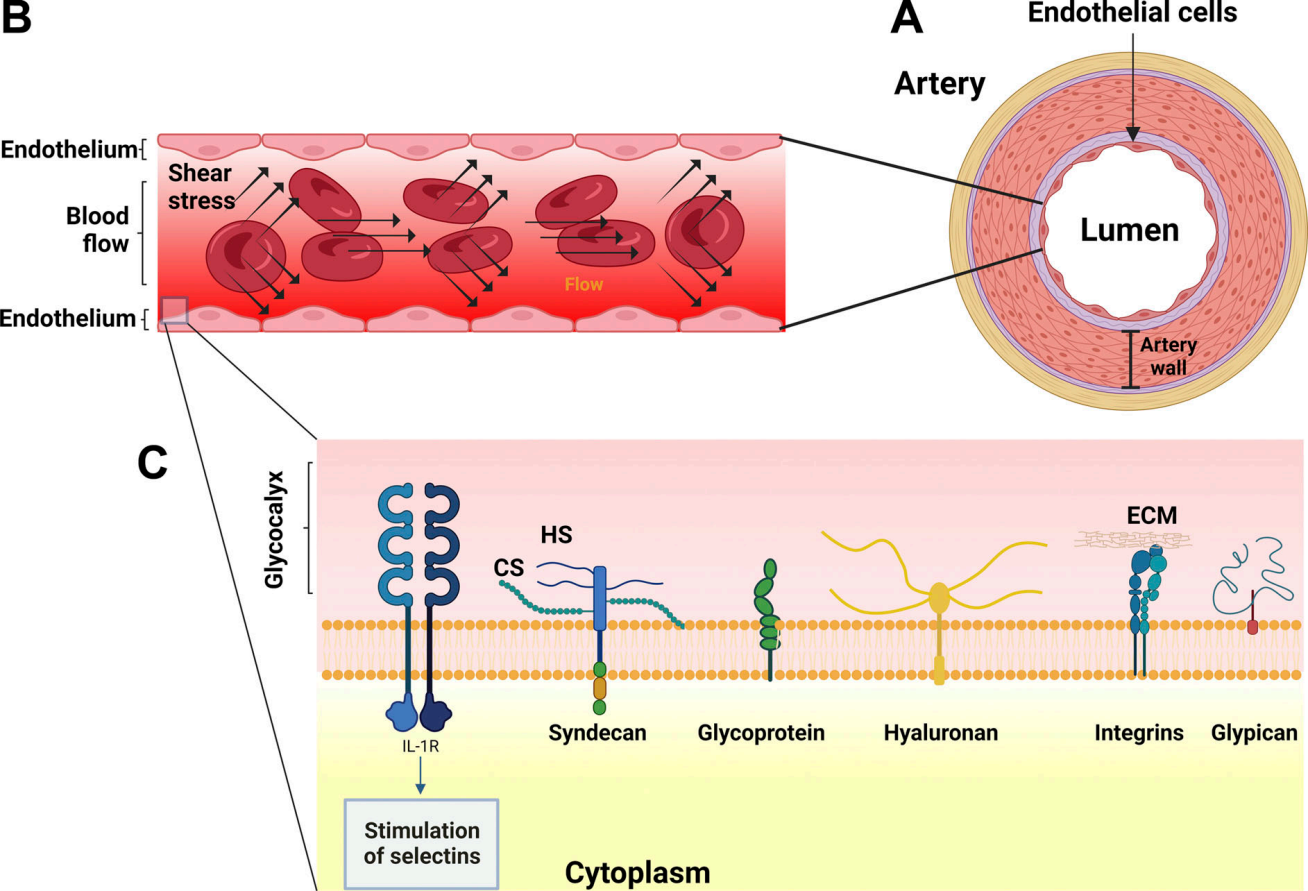
**Shear stress mechanotransduction in endothelial cells and the structure and function of the glycocalyx. (A)** The endothelial cell layer covers the internal surface of the arteries and releases substances to the artery wall that control vascular relaxation and contraction. **(B)** Blood flow and schematic representation of the mechanical forces inside the artery. **(C)** Structure and the main components of the endothelial glycocalyx. The glycocalyx is composed of proteoglycans, with long glycosaminoglycan side-chains and glycoproteins (with short-branched carbohydrate side chains). The syndecan family and glypican family are transmembrane HS proteoglycans and are believed to govern the transvascular distribution of fluid and protein. It is also known that cellular endothelial stimulation by cytokines such as IL-1R express selectins. The activity of integrins is influenced by glycans through glycosylation events and coordinates ECM interactions. Abbreviations: HS, heparan sulfate; CS, chondroitin sulfate; ECM, extracellular matrix; IL-1R, interleukin-1.

The endothelial glycocalyx contains membrane-binding proteoglycans formed by glycosaminoglycan (GAG), and the presence of hyaluronan and glycoproteins (Van Teeffelen et al., 2007; Fig. 2). The GAGs are negatively charged polysaccharide chains (Prydz, 2015), enabling binding to various growth factors and cytokines (Reitsma et al., 2007). Hyaluronan, mainly located in the luminal part of the glycocalyx, binds to surface receptors such as CD44 (Lennon and Singleton, 2011; Liu et al., 2013). Syndecans are central to maintaining endothelial cell homeostasis (Tkachenko et al., 2005). Endothelial cells express both E-selectin and P-selectin through distinct mechanisms (Sperandio, 2006; Fig. 2). Integrins, transmembrane receptors, facilitate platelet-endothelial cell interactions within the extracellular matrix (Bombeli et al.,1998; Rüegg and Mariotti, 2003; Fig. 1).

The regulation of vascular tone and the facilitation of endothelial–blood cell interactions, in response to shear stress, are the main roles of the glycocalyx (Fig. 2). When the flow becomes multidirectional, the GAG’s structure undergoes modifications, all connected to the cytoskeleton through linker molecules (Tarbell et al., 2014). Moreover, NO is produced, promoting the relaxation of smooth muscle cells and inducing vasodilation (Rubanyi et al., 1986).

The negatively charged endothelial glycocalyx acts as an electrostatic barrier regulating the permeability of water, small molecules, and oxygen, while also facilitating selective sodium ion buffering (Chappell et al., 2009; Fig. 2). In cases of inflammation, various cytokines activate the expression of tissue factors on endothelial cells, leading to the conversion of fibrinogen to fibrin (Prior et al., 2017; Fig. 2). Importantly, the glycocalyx plays an essential physiological role in recruiting leukocytes to sites of infection, where cell adhesion molecules such as integrins and immunoglobulin remain concealed within the glycocalyx (Iba and Levy, 2019).

### The endothelial caveolae and mechanotransduction events

The endothelial caveolae serve as functional units responsible for sensing and transducing blood flow-induced changes into biochemical signals. Caveolin-1, a protein constituting caveolae, shows significant expression after an increase in shear stress, and its absence leads to the remodeling of vascular abnormalities (Yu et al., 2006). Interruption of blood flow results in a marked decrease in mechanosignaling within endothelial cells, as observed in genetically engineered caveolae-depleted intact lungs (Noel et al., 2013). The exact mechanism by which caveolae function as mechanosensors remains unclear, but it is believed to be functionally linked to other mechanosensing molecules such as platelet endothelial cell adhesion molecule-1 (PECAM-1; Noel et al., 2013; Chatterjee and Fisher, 2014).

Hence, through these mechanotransducing events, endothelial cells transform diverse hemodynamic stimuli into biochemical or electrical signals, ultimately triggering cellular physiological or pathological responses. The endothelial cell surface is equipped with a range of specialized mechanosensors, including integrins, stretch-activated channels, PECAM-1, the membrane lipid bilayer, junctional proteins, tyrosine kinase receptors, caveolins, and the glycocalyx (Zhou et al., 2014).

Through adaptor molecules such as Shc (adaptor protein p66), Grb2 (growth factor receptor-bound protein 2), and Sos (son of sevenless), mechanical stimulation triggers a cascade of signaling pathways. Consequently, this modulation of signaling pathways leads to a differential expression of genes related to endothelial cell morphology and functions (Chiu and Chien, 2011).

### Endothelial cell proteins and their influence on mechanotransduction

A cascade of signaling, responsible for various changes in cell behavior, is triggered during the process of mechanotransduction. The concentration of cytoskeletal elements at adhesion and intercellular contact sites generates stress forces, serving as the major inducer of mechanical signaling. Mechanotransduction in endothelial cells is mediated by complex protein elements such as PECAM1, VE-cadherin, and VEGFR2, inducing changes in the endothelial phenotype in response to mechanical stimuli (Shihata et al., 2016).

Blood flow activates PECAM1-associated mechanotransduction through the GTP1 exchange factor known as TIAM1, leading to the activation of Rac1 GTPase in endothelial cells (Collins and Tzima, 2014). The activation of Rac1 triggers the NF-κB signaling pathway and the generation of ROS, whereas the membrane sphingosine-1-phosphate receptor 1 also can influence the cellular response to the direction of flow (Jung et al., 2012; Fig. 1).

## Mechanosensitive ion channels

Endothelial cells possess membrane-associated ion channels responsive to mechanical forces, sharing similarities with those observed in simpler life forms (Martinac et al., 1987; Sachs, 1988). Cultured endothelial cells have specific and nonspecific cation channels, and interactions among these channels are mediated through the consequences of their opening (Adams et al., 1989). For instance, the activation of potassium channels typically leads to cellular hyperpolarization, resulting in increased calcium influx via an activated calcium channel (Lückhoff and Clapham, 1992). Conversely, depolarization diminishes calcium influx, affecting NO release and vasodilation (Lückhoff and Busse, 1990). Endothelial cells exhibit common stretch-activated nonspecific cation channels (Lansman et al., 1987), contrasting with shear stress-activated potassium channel activity (Olesen et al., 1988).

The so-called Piezo1 is another mechanoreceptor and a mediator of shear stress associated with calcium mobilization. Depending on the intensity of shear stress, it enhances calcium flux, initiating protease activities and partial remodeling of endothelial cell polarity. Notably, in cultured endothelial cells from mice lacking Piezo1, there is a failure to align, respecting the flow in response to the impacting blood flow (Ranade et al., 2014a; Fig. 2).

**Figure 3.**
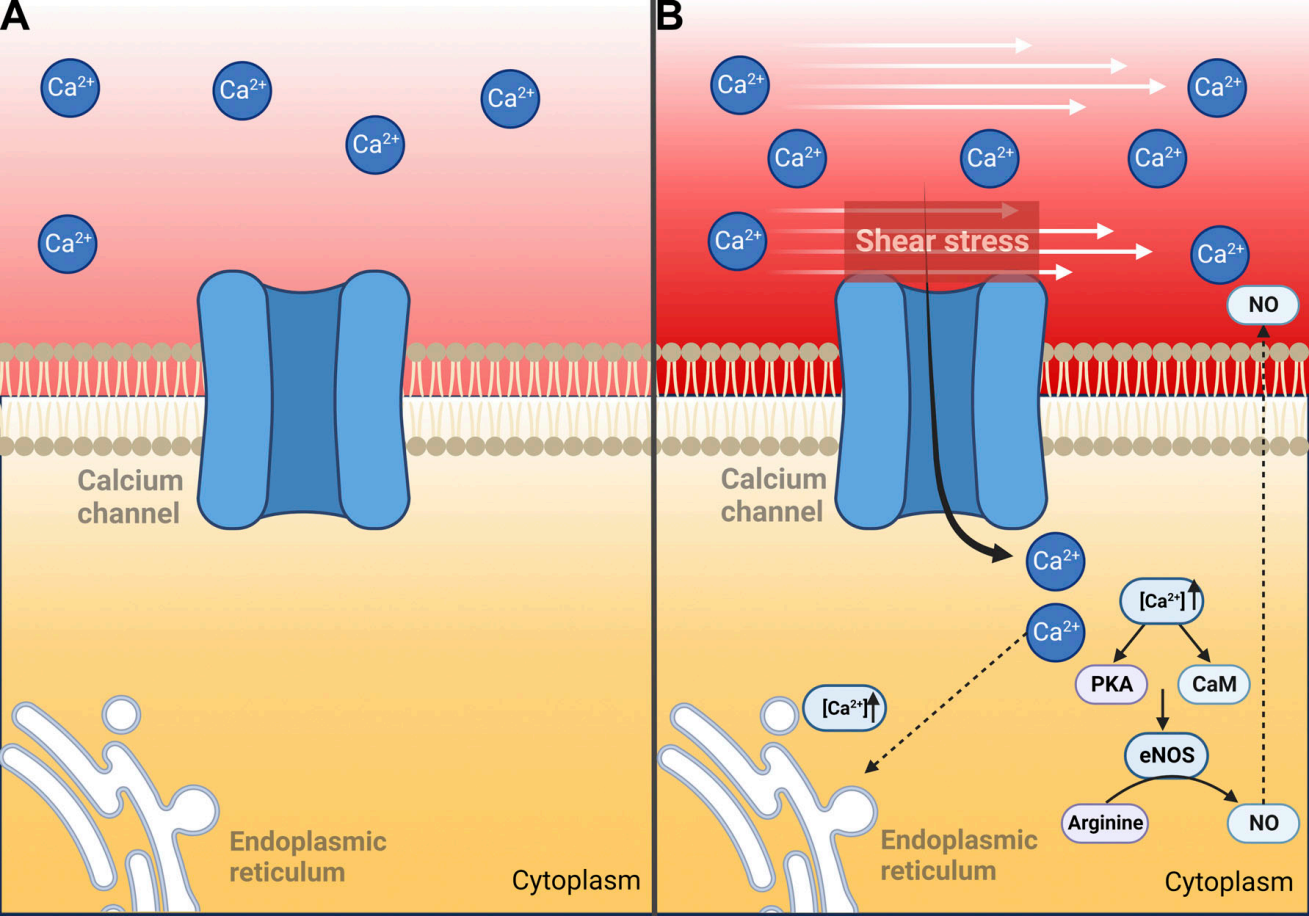
**Mechanosensitive calcium channels. (A)** Inactive mechanosensitive channels, without mechanical stimuli. **(B)** The active mechanosensitive channels that respond to membrane tension by altering their conformation between an open state and a closed state. eNOS increases NO expression following calcium increase through the CaM and PKA pathway. NO can freely diffuse. Furthermore, the increase in intracellular calcium concentration enhances endoplasmic reticulum calcium signaling. Abbreviations: CaM, calmodulin; PKA, protein kinase A; eNOS, endothelial nitric oxide synthase; NO, nitric oxide.

In this context, Piezo channels have been identified as stretch-sensitive, directly activated by applying negative pressure to the membrane (Coste et al., 2012). Additionally, Piezo has been shown to be activated by touch, such as poking the cells with a blunt probe (Ranade et al., 2014b; Fig. 3). The first evidence suggesting that Piezo channels are sensitive to shear stress emerged from experiments using a perfusion tube positioned in close proximity to the cell to record shear-induced currents (Ranade et al., 2014b). Increasing wall shear stress induced an elevated whole-cell Piezo current. Indeed, cell deformation induced by fluid shear stress appears to be sufficient to activate Piezo. The flow sensitivity of Piezo1 was demonstrated by a significant increase in current in response to an increased perfusion rate. However, it is not clear from these experimental approaches whether this mechanical stimulus reliably activates the channels (Li et al., 2014).

In response to shear stress, endothelial cells undergo a process involving the separation of β-catenin of TRPV4 channels, leading to TRPV4 channels being relocated at the focal adhesions in the basal membrane. This relocation subsequently increases endothelial permeability and induces calcium-mediated cytoskeletal remodeling (Baratchi et al., 2017a).

### Vascular ion channel activated by stretch

In many cells from a great variety of organisms, this type of mechanotransducing ion channels has been identified (Sachs, 1988; Fig. 3). Among these cell types, stretch-activated ion channels are present in skeletal muscle (Guharay and Sachs, 1984), renal tubular epithelium (Sackin, 1987), as well as in vascular endothelial cells (Lansman et al., 1987). These stretch-sensitive channels specifically respond to cations and exhibit a conductance of ∼40 pS, characterized by two components controlling the aperture of the channel. An important consequence of channel activation is the influx of calcium, leading to cell depolarization (Fig. 3). Consequently, this influx of calcium can have profound effects on multiple biological responses to hemodynamic forces.

Variations of stretch-sensitive channels are activated by calcium, as seen in mechanosensitive potassium ion channels, playing a role in osmotic swelling and volume regulation (Davies, 1995). In nonvascular cells, other variations consist of the side-by-side presence of stretch-inactivated potassium-selective channels, which seem to play a role in functional neurons. The physiological property of endothelial cells to respond to stretch and shear stress–activated channels may be associated with significant fluctuations in hemodynamic forces (Fig. 3). During disturbed laminar flow, as in the cardiac cycle (Davies, 1989), the shear stretch generated by hemodynamic changes activates synergistic or antagonistic effects capable of modifying cell polarity and, therefore, the local tone at such endothelial sites (Davies, 1993).

### Focal adhesions as mechanotransducers

The role of focal adhesions has been extensively studied at sites of cytoskeletal-linked mechanotransduction where cell tension is transmitted through flow. Despite these sites being considered relatively inert (Geiger et al., 1984), it is now known that these sites may exhibit protein kinase activity quite similar to that expressed in viral oncogenes (Horvath et al., 1990; Turner and Miller, 1994) and protein kinase C (Turner and Burridge, 1991), implicating them in cell signaling.

## Mechanotransduction and NO regulation

Endothelial mechanotransduction produces NO since the mechanical stimulus exerted on endothelial cells by the flowing blood serves as a potent signal to activate endothelial-derived NO synthase (eNOS), leading to NO production (Hill and Meininger, 2012). Increased shear stress on the endothelial cells correlates with enhanced eNOS activity, resulting in the release of NO. NO then stimulates receptors in arterial smooth muscle cells through a G-protein coupled cascade, ultimately causing receptor desensitization as part of the cascade initiation. Moreover, the effect of this process is the endocytosis of NO receptors. As the concentration of NO increases, the rate of endocytosis exceeds the rate of receptor reappearance on the cell surface, further contributing to the reduction in the content of membrane receptors (Bellamy et al., 2000). As the receptors become saturated, the concentration of NO in the vessel lumen increases. This excess NO is then scavenged by hemoglobin, ultimately leading to vasoconstriction (Olson et al., 2004).

## Liver and endothelial cells

**Figure 4.**
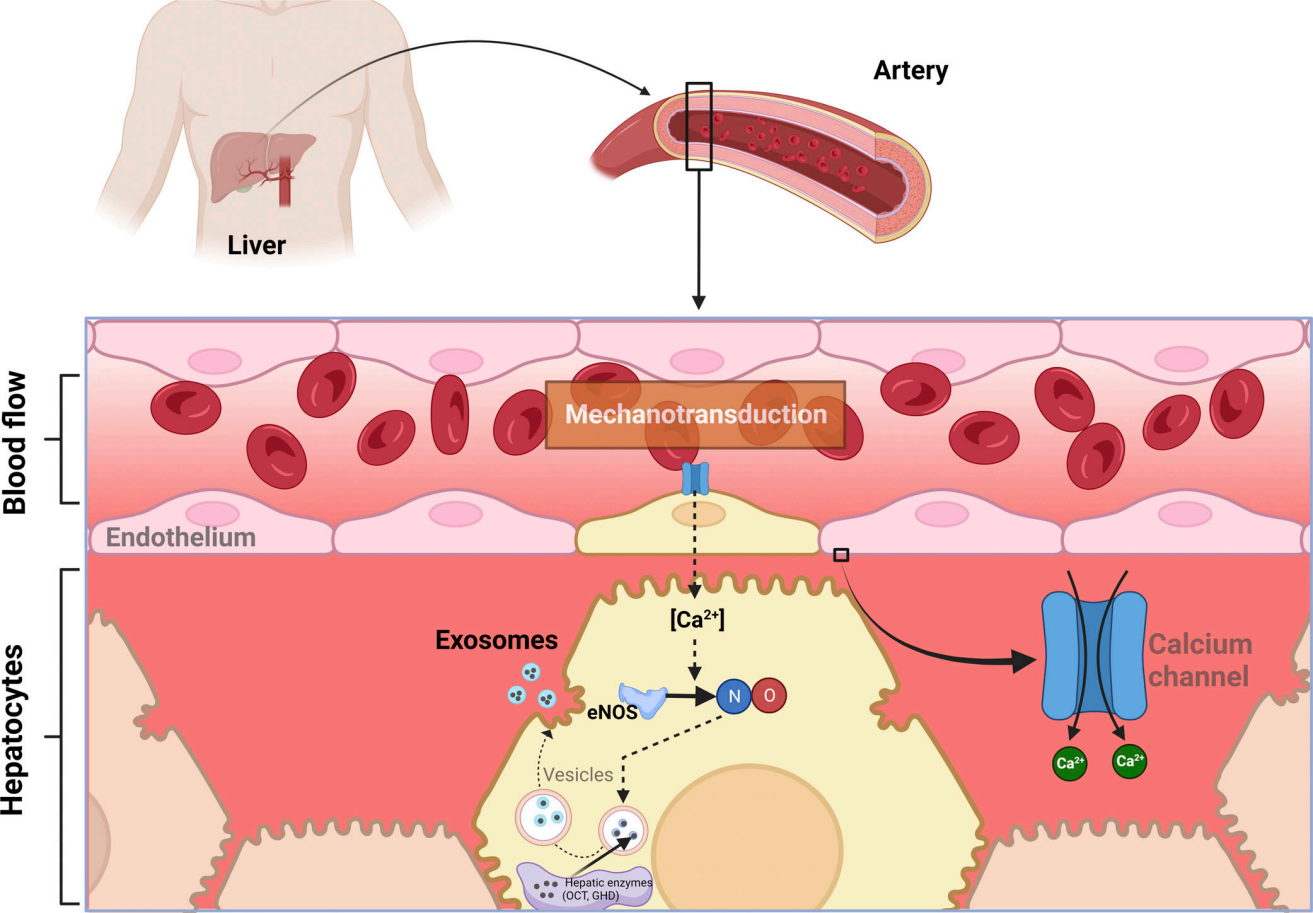
**Proposed mechanisms involved in shear stress (mechanotransduction)-mediated control of liver enzyme release.** We find that liver enzyme release is primarily controlled by extracellular calcium entry, mainly mediated by stretch-sensitive calcium channels. Endothelial-mediated mechanotransduction in liver enzyme release is significantly altered by modifying the glycocalyx carbohydrate components, the directionality of perfusing flow rate, and the participation of NO and malondialdehyde. These modifications lead to changes in the intracellular distribution of these enzymes. Therefore, flow-induced shear stress may offer finely tuned control of released hepatic enzymes through mediation by the endothelial glycocalyx. This provides evidence for a putative biological role of enzyme release, which appears to occur through exocytosis (output of exosome vesicles) from the hepatocytes. In this process, a pool of enzymes, independent of their metabolic functions, is destined to be released into the extracellular medium.

Endothelial cells constitute a significant subset of non-parenchymal cells, with the majority of them being non-continuous endothelial cells known as liver sinusoidal endothelial cells (LSECs; Fig. 4). The hepatic endothelium exhibits distinctive structural and functional characteristics (Poisson et al., 2017). Firstly, it is discontinuous and features open pores, accompanied by the absence of a basement membrane and tight junctions. Secondly, the liver sinusoid demonstrates a high capacity for endocytosis, frequently exposed to elevated levels of macromolecules, toxins, and waste products. Lastly, LSECs possess immunological functions (Fig. 4).

Under pathological conditions, LSECs undergo phenotypic changes at the molecular and cellular levels. These phenotypic alterations in LSECs participate in the progression of nonalcoholic fatty liver disease (NAFLD; Hammoutene and Rautou, 2019). Moreover, apart from LSECs, other types of hepatic endothelial cells exist, such as periportal and pericentral cells in mice (Xiong et al., 2019).

The synergistic effects of mechanotransduction and other biochemical signaling on LSEC function are still under investigation, with the aim of developing integrated strategies and agents that can enhance the clinical outcomes of chronic liver disease.

## Liver enzyme release, endothelium, and mechanotransduction

To investigate inter-organ communication and the potential signals involved, we utilized a rat model of partial hepatectomy. Our findings revealed that following a 70% partial hepatectomy in healthy rats, there is a significant and temporary increase in serum activities of certain liver enzymes known as “escape” enzymes, without any evidence of liver damage (Díaz-Juárez et al., 2006). Interestingly, we demonstrated that this increase in serum enzyme activities after partial hepatectomy is linked to liver hemodynamic changes in the blood flow to liver mass ratio (Fig. 4). We observed that the release of enzymes from perfused rat liver was reversible and not associated with the production of extrahepatic factors (Díaz-Juárez et al., 2006).

### Roles of calcium, NO, and stretch-sensitive calcium channels on liver enzyme release

We made additional discoveries regarding the release of liver enzymes, highlighting their dependence on mobilizing calcium, probably by stretch-sensitive calcium channels, and increasing NO production (Figs. 3 and 4; Díaz-Juárez and Hernández-Muñoz, 2011). Furthermore, the putative role of mechanotransducing events in liver enzyme release was also suggested by the impact of increased shear stress (by augmenting the viscosity of the perfusion medium), which selectively influenced the release of the tested enzymes (Díaz-Juárez and Hernández-Muñoz, 2011; Fig. 4).

Our investigation into in vivo protein synthesis inhibition revealed that the release of liver enzymes is indeed influenced by the de novo synthesis of endothelial glycocalyx protein components. Additionally, the released enzymes are confined to a liver “pool” (Díaz-Juárez and Hernández-Muñoz, 2017; Fig. 4). Moreover, the dependency of liver enzyme release on extracellular calcium entry seems to be mediated by stretch-sensitive calcium channels (Fig. 3). The involvement of endothelial-mediated mechanotransduction in liver enzyme release was further supported by modifying the glycocalyx carbohydrate components, the directionality of perfusing flow rate, and the participation of NO and malondialdehyde, resulting in alterations in the intracellular distribution of these enzymes (Díaz-Juárez and Hernández-Muñoz, 2017).

The enzymatic removal of carbohydrate residues would have differential effects on the release of cytoplasmic enzymes and mitochondrial enzymes, specifically OCT and GDH activities. Interestingly, pretreatment with heparanase, an enzyme that removes heparan sulfates, demonstrated that heparan sulfates actively participate in mechanotransduction, leading to the production of NO/ROS and increased permeability (Dull et al., 2007). Furthermore, the integrity of the vascular endothelial cell glycocalyx plays a crucial role in regulating the mediation of coronary flow on heart glycolytic flux (Suárez and Rubio, 1991).

The increased intracellular calcium concentration is inhibited by gadolinium, indicating the participation of extracellular calcium through stretch-sensitive channels (Munevar et al., 2004; Fig. 3). Through our experimental approaches, we have demonstrated that the release of liver enzymes is associated with extracellular calcium dynamics, primarily influenced by shear stress since there was no enhancement of calcium entry in liver preparations not subjected to physical forces (Díaz-Juárez and Hernández-Muñoz, 2017). The calcium release channels within the intracellular space are targeted by mechanisms of metabolic control during the proliferative response following 70% partial hepatectomy and may contribute to modified intracellular calcium dynamics (Díaz-Muñoz et al., 1998; Fig. 4).

In many biological models, it has been established that cell stretch induces ongoing physical events in cell signaling in the process of mechanotransduction (Trepat et al., 2007). In addition, the cytoskeleton organization and stabilization of focal adhesions are influenced by the interaction of cells with their substrate (Discher et al., 2005). Therefore, the available data indicate that mechanotransduction-linked effects impact hepatocytes, which respond with a controlled release of hepatic enzymes into the bloodstream not associated with liver oxidative stress (Contreras-Zentella and Hernández-Muñoz, 2016; Fig. 4).

### Roles of mechanotransduction, exosome formation, and liver enzyme release

Recently, there has been growing evidence highlighting the participation of mechanical stress in the exocytosis of membrane proteins, as well as in the generation of “exosomes” that carry regulatory molecules (Baratchi et al., 2017b). These eukaryotic cell–produced exosomes can transport proteins and RNA molecules from the original cell depending on the cellular stress conditions. They have the potential to regulate endothelial responses to these stress conditions (de Jong et al., 2012). Furthermore, in vitro experiments have shown that shear stress–mediated exocytosis of membrane molecules sensitizes endothelial cells, as observed in the case of P2X receptors responding to ATP and TRPV4 to pharmacological agonists (Baratchi et al., 2016).

In our in vitro model using liver preparations, we have discovered that soluble enzymes are released from hepatic tissue at a constant rate. A significant portion of this enzyme output occurs through an exocytosis mechanism in the form of vesicles that we were able to isolate using ultracentrifugation. The amount of released enzymes in exosomes varies depending on their intracellular location and is influenced by the metabolic energy and cytoskeleton integrity of the hepatocyte (unpublished data), as depicted in Fig. 4. We believe that the main function of maintaining a constant efflux of enzymatic proteins into the bloodstream (Fig. 4) is to establish a signaling system between organs, capable of influencing the metabolism of the target organ. Apart from their catalytic function, liver enzymes may possess additional properties, constituting a part of a complex signaling system that is evident in the metabolic adjustments required to compensate for the sudden surgical loss of functional hepatic mass. Actually, the intravenous administration of soluble liver enzymes, such as OCT, produces pharmacological effects in rats subjected to 30% partial hepatectomy (Díaz-Muñoz and Hernández-Muñoz, 2010).

## Concluding remarks

A new kind of cell signaling emerges as a result of the mechanical activation of stretch-gated ion channels. At the molecular level, external perturbations such as shear, compressive, or tensile stress exert mechanical forces that modulate contractility by regulating RhoA activity in conjunction with Rho-associated serine/threonine kinase (ROCK) and integrins (Fig. 1). The mechanism of mechanotransduction in endothelial cells also involves complex protein elements, such as PECAM1, VE-cadherin, and VEGFR2, which induce changes in the endothelial phenotype in response to mechanical stimuli. The endothelial glycocalyx, primarily composed of membrane-binding proteoglycans and glycosaminoglycans, plays a crucial role in NO production as a biological response to mechanotransduction (Fig. 2). Moreover, liver sinusoidal endothelial cells (LSECs) constitute a major subpopulation of non-parenchymal cells that line the hepatic sinusoids.

We have demonstrated that hemodynamic changes, likely acting on the endothelium as well as the extracellular matrix and hepatocytes, play a crucial role in stimulating the release of enzymes from the intact liver, which relies heavily on extracellular calcium influx (Fig. 3), representing a primary response to hemodynamic changes and may play an important role in participation. This response is potentially mediated through the modulation of various biochemical pathways, including shear stress-induced exocytosis, cellular energy availability, and intracellular trafficking (Fig. 4). The release of liver enzymes, thus, emerges as a novel phenomenon controlled by hemodynamic changes and could be implicated in several human pathologies. These findings also raise the possibility of evaluating the clinical implications for patients receiving medication that affects hemodynamics and/or systemic blood pressure.

## Data Availability

No new data were generated in support of this study.

## Data availability

No new data were generated in support of this study.
